# Serum Angiopoietin-like Protein 3 Levels Are Associated with Endothelial Function in Patients with Maintenance Hemodialysis

**DOI:** 10.3390/life14010018

**Published:** 2023-12-21

**Authors:** Tzu-Chiang Wu, Bang-Gee Hsu, Chiu-Huang Kuo, Chih-Hsien Wang, Jen-Pi Tsai

**Affiliations:** 1 Department of Internal Medicine, Hualien Tzu Chi Hospital, Buddhist Tzu Chi Medical Foundation, Hualien 97004, Taiwan; 103311117@tzuchi.com.tw (T.-C.W.); geelily@tzuchi.com.tw (B.-G.H.); hermitkuo@tzuchi.com.tw (C.-H.K.); wangch33@tzuchi.com.tw (C.-H.W.); 2 Division of Nephrology, Hualien Tzu Chi Hospital, Buddhist Tzu Chi Medical Foundation, Hualien 97004, Taiwan; 3 School of Medicine, Tzu Chi University, Hualien 97004, Taiwan; 4 School of Post-Baccalaureate Chinese Medicine, Tzu Chi University, Hualien 97004, Taiwan; 5 Division of Nephrology, Department of Internal Medicine, Dalin Tzu Chi Hospital, Buddhist Tzu Chi Medical Foundation, Chiayi 62247, Taiwan

**Keywords:** endothelial function, angiopoietin-like protein 3, hemodialysis, digital thermal monitoring test, vascular reactivity index

## Abstract

Angiopoietin-like protein 3 (ANGPTL3) plays an important role in lipid and lipoprotein trafficking and metabolism and is positively correlated with cardiovascular disease. Our objective was to evaluate the association between serum ANGPTL3 levels and endothelial function in patients on maintenance hemodialysis (MHD). We enrolled 116 patients on MHD and obtained their blood test results from their medical records. Using a noninvasive digital thermal monitor, we determined the vascular reactivity index (VRI) as a measure of endothelial function. Serum ANGPTL3 concentration was measured by a commercial-enzyme-linked immunosorbent assay. Vascular reactivity was classified as poor in 17 (14.7%) patients, intermediate (1.0 ≤ VRI < 2.0) in 50 (43.1%) patients, and high (VRI ≥ 2.0) in 49 (42.2%) patients. Serum levels of ANGPTL3 (*p* < 0.001) and alkaline phosphatase (ALP, *p* = 0.025) increased significantly as the VRI decreased. The log-transformed serum ALP (log-ALP, *r* = −0.187, *p* = 0.045) and log-ANGPTL3 (*r* = −0.319, *p* < 0.001) showed a negative correlation with the VRI on univariate linear regression analysis. A significant negative correlation was found between log-ANGPTL3 and VRI (*p* < 0.001) on multivariate stepwise linear regression analysis. The findings of our investigation showed that, in patients with MHD, the ANGPTL3 concentration had a negative correlation with the VRI.

## 1. Introduction

In patients on maintenance hemodialysis (MHD) for end-stage renal disease (ESRD), cardiovascular disease (CVD) is significantly frequent [1]. In the United States, in 2018, CVD was a comorbidity in 56.2%, which represents 76.5% of all deaths among patients receiving MHD [2]. Endothelial dysfunction (ED) has recently been shown to be a critical factor in the development of atherosclerosis and CVD [3].

A healthy vascular endothelium is essential in order to adjust blood flow and vascular resistance. An imbalance in the production of vasoconstrictors and vasodilators in the endothelial region of the vessel is called ED [4] and is reported to exist in patients with early-stage chronic kidney disease (CKD) based on vasodilatation dependent on the endothelium in the brachial artery. Moreover, the worsening of ED has been related to the progression of CKD to ESRD [5,6]. Based on the connection between atherosclerosis and ED, as well as the reversibility of early atherosclerotic lesions, numerous biomarkers have become available for the early detection and prevention of CAD and ED [7]. However, only a few indicators are ideal for clinical application [8].

The liver-exclusive angiopoietin-like protein 3 (ANGPL-3) inhibits lipoprotein lipase in conjunction with angiopoietin-like 8 [9]. It was shown that people with a loss-of-function mutation in ANGPL3 had relatively low levels of serum triglycerides, total cholesterol, high-density lipoprotein cholesterol, and low-density lipoprotein cholesterol (LDL-C). This result was in line with a comparatively low risk of CVD caused by atherosclerosis. [10,11]. Using antiANGPL3 monoclonal antibodies, animals and humans also showed decreased lipid fractions and risk of CVD [12]. Additionally, ANGPL3 levels in patients on chronic MHD are two times greater than in age- and gender-matched healthy controls [13]. In diabetic rats, treatment with the dipeptidyl peptidase-4 (DPP-4) inhibitor vildagliptin led to a significant suppression of ANGPL3 (*Angptl3*) expression and alleviated ED in the aorta [14]. However, the relationship between serum ANGPL3 levels and ED remains unclear. With the hypothesis that ANGPL3 contributes to dyslipidemia and causes ED, we conducted this study to determine the correlation between ANGPL3 and ED in patients on MHD. A further question is the role of serum ANGPL3 level as an alternative biomarker for endothelial function.

## 2. Materials and Methods

### 2.1. Participants

With approval from the Hualien Tzu Chi Hospital’s research ethics committee (IRB109-122-C), we enrolled patients on MHD who were older than 20 and who were receiving regular HD for longer than three months between January 2021 and March 2021. Individuals who were bedridden; had active infections, cancer, heart failure, or amputated limbs; or refused to give informed consent were excluded. Finally, 116 patients on chronic MHD were enrolled in this study. Medical records were examined to determine the basic clinical characteristics of the patient, length of MHD treatment, and medical history, which included diabetes mellitus (DM), hypertension (HTN), and medications taken.

### 2.2. Measurement of Body Mass Index and Biochemistry

The body mass index (BMI) was determined by measuring the square height (m^2^) and body weight (kg) followed hemodialysis. [15]. Before initiating hemodialysis, each patient had a blood sample of about 5 milliliters obtained. An autoanalyzer (Siemens Advia 1800; Siemens Healthcare, Henkestr, Erlangen, Germany) was used to measure the hemoglobin level (Sysmex SP-1000i, Sysmex American, Mundelein, IL, USA) and centrifuge the remaining blood sample for biochemical analyses. The dialysis clearance was measured using the urea kinetic model, which includes the urea reduction ratio and the fractional clearance index for urea (Kt/V). The serum levels of intact parathyroid hormone (iPTH) (Diagnostic Systems Laboratories, Webster, TX, USA) and ANGPTL3 (R&D Systems, Inc., Minneapolis, MN, USA) were measured using commercially available assay kits. [16,17].

### 2.3. Endothelial Function Measurements

Following a night’s fasting and the cessation of alcoholic beverages and caffeine, endothelial function was assessed in all subjects using an FDA-approved device (VENDYS-II; Endothelix, Inc., Houston, TX, USA). The vascular reactivity index (VRI) was calculated using methods that had been previously published. [15,18,19]. The patients’ VRI was categorized as poor (0 to <1.0), intermediate (1.0 to <2.0), or good (≥2.0).

### 2.4. Analytical Statistics

The Kolmogorov–Smirnov test was used to evaluate the continuous variables’ normal distribution. The results were displayed as mean ± standard deviation for continuous variables with a normal distribution, or median with interquartile range for skewed continuous variables. Such continuous variables as MHD duration and the levels of triglyceride, glucose, ALP, and iPTH among the VRI groups based on the results of the Kolmogorov–Smirnov test were compared using one-way analysis of variance for normal distribution or Kruskal–Wallis analysis for skewed distribution. Categorical variables were reported as numbers and percentages and compared among groups using the chi-square test. To ensure normality for additional analysis, log-transformed values of duration of MHD and triglyceride, glucose, ALP, and iPTH levels were obtained. The association between VRI and the clinical and biochemical characteristics of the patients on MHD was investigated using straightforward multivariate and linear stepwise regression analyses. By controlling for variables such as sex, age, and BMI; systolic and diastolic blood pressures; the presence of DM and HTN; MHD duration and Kt/V; and ANGPTL3 levels, the relationships between the ANGPTL3 level and poor vascular reactivity and vascular reactivity dysfunction—defined as both intermediate and poor vascular reactivity—were investigated using studies of logistic regression, both univariate and multivariate. The effect of ANGPTL3 level on dysfunctional and low vascular reactivity was first determined, and then the power was determined by displaying the receiver operating curve (ROC) and calculating the area under the curve (AUC). SPSS Version 19.0 for Windows (SPSS Inc., Chicago, IL, USA) was used to perform the analyses. A statistically significant *p* value was defined as <0.05.

## 3. Results

The clinical characteristics and prescription drugs taken by the study population (N = 116) are shown in Table 1. The VRI was good in 49 (42.2%), intermediate in 50 (43.1%), and poor in 17 (14.7%). The serum levels of ALP (*p* = 0.025) and ANGPTL3 (*p* < 0.001) were significantly higher in the poor and intermediate VRI groups than in the good VRI group. The coexisting diseases were DM in 59 patients (50.9%) and HTN in 67 patients (57.8%). The categories of patients did not differ substantially in terms of blood pressure, age, sex, duration of MHD, URR, Kt/V, presence of DM or HTN, or usage of medications for dyslipidemia and HTN. A total of 59 patients (50.9%) on MHD had DM and 67 patients (57.8%) on MHD had HTN, respectively. The groups did not differ substantially in terms of blood pressure, age, sex, duration of MHD, URR, Kt/V, causes of MHD, vascular access, presence of DM or HTN, or usage of drugs for dyslipidemia and HTN.

**Figure 1:**
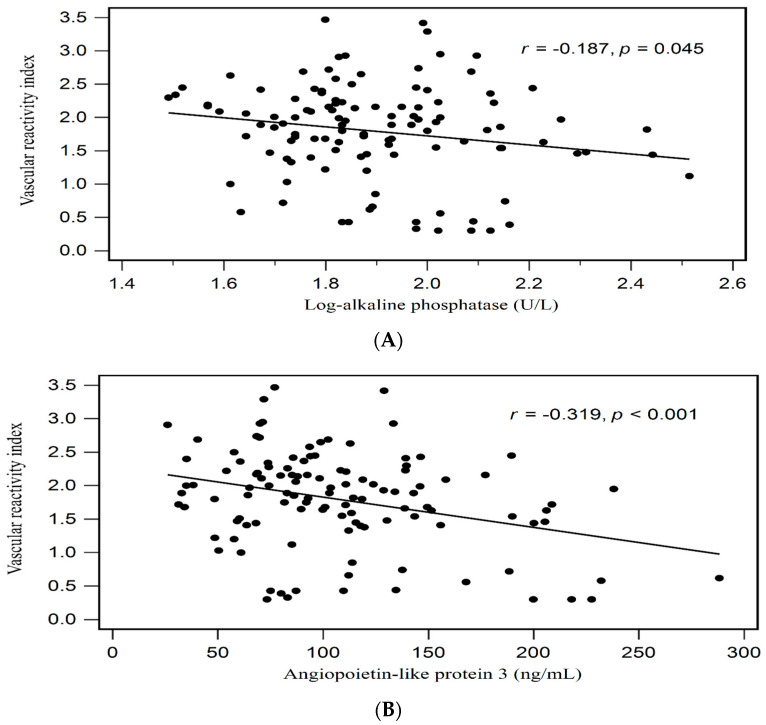
Relationships between VRI and (**A**) log-transformed alkaline phosphatase (log-ALP), and (**B**) ANGPTL3 among patients with MHD.

The correlations between clinical features and the VRI are displayed in Table 2. The serum level of ANGPTL3 (*r* = −0.319, *p* < 0.001) and log-ALP (*r* = −0.187, *p* = 0.045) showed a negative correlation with the VRI. The VRI was found to be independently correlated with the serum ANGPTL3 level (β = −0.319, adjusted R2 change = 0.094, *p* < 0.001) on multivariate forward stepwise linear regression analysis. Figure 1 shows the two-dimensional scatter plots of the VRI with the serum log-ALP and ANGPTL3 levels.

For further analysis by univariate and multivariate logistic regression analysis, MHD patients with poor and intermediate vascular reactivity were combined to indicate vascular reactivity dysfunction. This multivariate analysis was controlled for blood urea nitrogen, creatinine, total cholesterol, triglyceride, alkaline phosphatase, Kt/V, sex, age, body mass index, diabetes mellitus, hypertension, hemodialysis duration, and ANGPTL3. The results showed that ANGPTL3 was positively and independently associated with both poor vascular reactivity (odds ratio (OR) = 1.024, 95% confidence interval (CI) = 1.010–1.037, *p* = 0.001) and vascular reactivity dysfunction (OR = 1.015, 95% CI = 1.005–1.026; *p* = 0.005) (Table 3).

The ROC curve for predicting vascular reactivity dysfunction by ANGPTL3 revealed that the AUC was 0.635 (95% CI = 0.541–0.723, *p* = 0.0086) and poor vascular reactivity by ANGPTL3 demonstrated the AUC was 0.713 (95% CI = 0.622–0.793, *p* = 0.0019), respectively (Table 4).

## 4. Discussion

Serum ANGPL3 levels and endothelial function in MHD patients were found to be significantly correlated negatively in the current study. Mainly produced in the liver, the 70-kDa protein ANGPL3 has a physiological function that involves blocking lipoprotein and endothelium lipases, which impacts triglyceride hydrolysis. [9]. According to previous research, Ghiadoni et al. found that ED measured by flow mediated dilation method was lower in CKD patients compared with healthy participants and reduced more in MHD, which is indicative of inverse correlation between renal function and ED severity patients [20]. Research showed that serum ANGPL3 levels in MHD patients were two times greater than in those with chronic MHD compared with healthy people [13] and another study showed that these levels increased pre-dialysis to MHD patients [21]. The Birsighella Heart Study reported a strong correlation between the serum ANGPL3 levels and the suboptimal ankle-brachial index in older healthy patients, which could be an early biomarker for peripheral arterial disease [22]. From another study, Hastuda et al. reported that ANGPTL3 was positively associated with the carotid as well as femoral artery intima-media thickness, independent of age, sex, smoking status, BMI, lipid levels, systolic blood pressure, and insulin resistance index in healthy individuals [23]. Our research revealed a negative correlation between ANGPTL3 and the vascular reactivity index, which led us to hypothesize that ANGPL3 may be involved in modulating the structure or function of vascular walls. 

The relationship between ANGPL3 and endothelial function may have a theoretical explanation. The relatively low plasma LDL-C levels in individuals with loss-of-function mutations in ANGPL3 are widely recognized [11]. Moreover, inhibition of ANGPL3 expression in humans and mice was reported to decrease the plasma LDL-C levels [12]. Put differently, individuals with elevated ANGPL3 levels were shown to have elevated LDL-C levels; in an environment of oxidative stress, LDL-C has a greater propensity to become oxidized LDL-C (OxLDL). After attaching to the endothelial scavenger cell receptor (i.e., cell cluster differentiation 36), OxLDL may move the complex of endothelial nitric oxide synthase (eNOS) and caveolin from the caveolae on the cell membrane into the cytoplasm. Transduction of nitric-oxide-producing signals from the extracellular space is prevented by eNOS translocation, which inhibits eNOS activity and causes ED [3,24]. The inhibited eNOS activity further impairs endothelial relaxation, which can also result in ED. We examined the relationship between ANGPL3 and lipid profiles in this study using individual Pearson and Spearman correlation analyses, and we observed no significant association between ANGPL3 and total cholesterol (*r* = 0.023, *p* = 0.804) and triglyceride (*r* = 0.047, *p* = 0.620). Another theory is related to the nonlipid actions of ANGPL3, which could directly cause structural vascular damage. With its fibrinogen-like C-terminal domain, ANGPL3 can attach itself to the αvβ3 integrin in endothelial cells, which can lead to inflammation, foam cell production, and endothelial damage [25]. Furthermore, one study on rats with DM showed that administration of medications, such as the DPP-4 inhibitors, significantly improved endothelial function and decreased the ANGPL3 expression in the aorta [14]. However, while it found an adverse association between endothelial function and serum ANGPL3 level in patients with ESRD on MHD, our research did not identify a significant relationship between prescription medications and endothelial function. As far as we know, our work was the first to draw attention to the unfavorable correlation that exists between ANGPL3 and endothelial function in patients with ESRD as determined by a noninvasive digital thermal monitor. Nevertheless, additional research is required to determine the potential mechanisms by which ANGPL3 contributes to endothelial dysfunction.

According to the KDIGO (Kidney Disease Improving Global Outcomes) 2017 clinical practice guideline update for the diagnosis, prevention, and treatment of chronic kidney disease–mineral and bone disorder, serum level of calcium, phosphate, PTH and alkaline phosphatase were recommended monitoring since CKD stage 3a. In addition, in CKD stage 3a to G5D patients, serum PTH and alkaline phosphatase could be used to evaluate bone disease because abnormal values could predict underlying bone turnover. In addition, patients on MHD had associated HTN, hypocalcemia, hyperphosphatemia, high calcium–phosphate product, parathyroid hormone, arterial stiffness, and ED [26]. However, in our study, no significant correlation was found between the VRI and the history of HTN and serum levels of calcium, phosphate, calcium–phosphate product, and iPTH. Nevertheless, we found a negative association between ALP and the VRI. There has been evidence of the association between ALP and CVD risk. Lai et al. conducted a multicenter retrospective analysis and found that serum ALP, regardless of age, was related to the atherosclerosis index severity score in patients with coronary artery disease, particularly in those with ALP greater than 83 U/L and an inflammatory condition [27]. Another study by Ren et al. indicated that compared with the lowest ALP tertile group (lower than 68 U/L), people with acute coronary syndrome and the highest ALP levels (more than 80 U/L) had comparably significant risks for calcification, patchy calcification, and plaque burden evaluated by intravascular ultrasound, suggesting that ALP may function as a biomarker for calcification and plaque vulnerability [28]. In a meta-analysis study, Kunutsor and colleagues showed a 1.08-fold increased pooled relative risk for CVD per 1 standard deviation change in log-ALP level in the general populations [29]. Furthermore, in patients on MHD, serum ALP level, especially higher than 120 U/L, was remarkably correlated with the coronary calcification score but not with the markers of inflammation, malnutrition, or bone and mineral problems [30]. In accordance with our results, Chen et al. observed that among kidney transplant recipients, elevated serum ALP was linked to reduced vascular reactivity [31]. By further analysis with ROC curve, we found that the AUC, optimal cut-off values, sensitivity, and specificity of ALP in predicting vascular reactivity dysfunction and poor vascular reactivity were 0.625 (95% CI = 0.530–0.713, *p* = 0.017), 66 U/L, 71.64%, 51.02% as well as 0.665 (95% CI = 0.572–0.750, *p* = 0.015), 76 U/L, 76.47%, 59.60%. These investigations, along with our findings demonstrating an inverse relationship between serum ALP levels and VRI in MHD patients, point to the involvement of ALP in endothelial function regulation. We did not discover similar results, despite evidence suggesting that PTH and vascular reactivity were negatively correlated and that calcium x phosphate product may be related to carotid artery distention [26,32]. According to a previous study, patients on MHD had significantly poorer brachial artery flow-mediated dilation and a more severe degree of arterial stiffness after 36 months than after 12 months [33]. Our study revealed a tendency for an association between a longer duration of MHD and poor vascular reactivity. The average MHD duration for the good, intermediate, and poor VRI groups was 36.6, 44.28, and 51.36 months, respectively. However, after multivariable linear regression analyses, there were no significant differences in MHD duration among the three VRI groups. In order to ascertain the causal link between endothelial function and the duration of MHD in larger cohorts, future investigations should employ longitudinal approaches.

Forearm flow-mediated dilatation has been recently mentioned as the gold standard approach to measure endothelial function in patients with CKD [34] and has been verified in numerous outcome studies to have a strong connection with coronary function. However, its intra- and inter-operator variability is high. In the present study, we quantified endothelial function using a digital thermal monitoring device (VENDYS-II), which is an operator-independent, standardized, and noninvasive technique to assess peripheral vascular reactive hyperemia and had been previously demonstrated to have strong intrasubject variability and good connection to Doppler recordings of hyperemic blood flow velocity [35]. Moreover, the results of the digital thermal monitoring approach were found to be highly associated with the computed tomography angiography diagnosis of obstructive coronary artery disease [36]. Therefore, this approach to measuring endothelial function is reliable. 

Our research had certain limitations. Initially, we discovered a negative relationship between serum ANGPL3 level and endothelial function in patients on MHD. Based on the analysis of the available literature, we inferred that individuals undergoing MHD had increased levels of inflammation, oxidative stress and serum LDL-C, all of which impair endothelial relaxation. However, our analysis did not include C-reactive protein, oxidative biomarkers, and serum levels of LDL-C and OxLDL. In our future study, we will use these markers to validate our inference. Second, the outcomes of our study might have been biased because of the limited sample size. Therefore, our next task will be to enlist additional research participants.

## 5. Conclusions

We found that the serum ANGPL3 level was negatively correlated with the VRI and played a central role in ED in patients on MHD. It might be a promising biomarker for the early evaluation of endothelial function in this population.

## Figures and Tables

**Table 1:** Clinical features of MHD patients classified according to measures of vascular reactivity index.

Characteristics	All Participants (*n* = 116)	Good Vascular Reactivity (*n* = 49)	Intermediate Vascular Reactivity (*n* = 50)	Poor Vascular Reactivity (*n* = 17)	*p* Value
Vascular reactivity index	1.79 ± 0.72	2.42 ± 0.37	1.62 ± 0.25	0.49 ± 0.18	<0.001 *
Age (years)	60.54 ± 13.11	58.18 ± 11.80	62.61 ± 14.80	61.25 ± 10.78	0.238
Female, *n* (%)	49 (42.2)	22 (44.9)	18 (36.0)	9 (52.9)	0.419
Diabetes mellitus, *n* (%)	59 (50.9)	26 (53.1)	27 (54.0)	6 (35.3)	0.379
Hypertension, *n* (%)	67 (57.8)	28 (57.1)	30 (60.0)	9 (52.9)	0.873
ANGPTL3 (ng/mL)	107.83 ± 50.64	92.20 ± 36.12	109.25 ± 49.79	148.72 ± 66.30	<0.001 *
Pre-MHD body weight (kg)	67.06 ± 15.05	67.55 ± 15.03	68.59 ± 16.06	61.13 ± 10.78	0.202
Post-MHD body weight (kg)	64.43 ± 14.55	64.95 ± 14.51	65.88 ± 15.42	58.68 ± 10.92	0.201
Body mass index (kg/m^2^)	25.14 ± 4.49	25.25 ± 4.50	25.49 ± 4.65	23.77 ± 3.95	0.388
MHD duration (months)	42.66 (25.92–79.71)	36.60 (24.78–85.14)	44.28 (23.25–100.50)	51.36 (37.26–78.24)	0.523
Systolic blood pressure (mmHg)	148.58 ± 28.62	151.45 ± 27.16	148.34 ± 28.85	141.00 ± 32.25	0.434
Diastolic blood pressure (mmHg)	80.76 ± 15.51	82.39 ± 14.67	79.90 ± 14.86	78.59 ± 19.81	0.603
Hemoglobin (g/dL)	10.24 ± 1.29	10.22 ± 1.23	10.34 ± 1.34	10.30 ± 1.35	0.976
Albumin (g/dL)	4.17 ± 0.46	4.25 ± 0.47	4.12 ± 0.44	4.12 ± 0.48	0.346
Total cholesterol (mg/dL)	157.13 ± 38.99	153.78 ± 38.72	159.36 ± 38.87	160.24 ± 41.78	0.732
Triglyceride (mg/dL)	127.00 (87.50–206.50)	127.00 (84.00–206.50)	127.50 (87.75–209.25)	114.00 (86.00–176.50)	0.948
Glucose (mg/dL)	127.50 (101.50–185.25)	120.00 (102.00–189.00)	136.50 (103.75–193.00)	116.00 (80.50–153.00)	0.328
Alkaline phosphatase (U/L)	74.00 (59.00–104.00)	66.00 (56.00–96.00)	75.00 (58.00–107.50)	95.00 (73.50–128.00)	0.025 *
Blood urea nitrogen (mg/dL)	60.20 ± 14.83	62.04 ± 13.46	58.88 ± 14.90	58.76 ± 18.44	0.524
Creatinine (mg/dL)	9.31 ± 2.14	9.76 ± 2.19	9.12 ± 1.97	8.61 ± 2.34	0.109
Total calcium (mg/dL)	9.07 ± 0.76	9.06 ± 0.74	9.05 ± 0.76	9.16 ± 0.84	0.872
Phosphorus (mg/dL)	4.88 ± 1.42	5.07 ± 1.44	4.77 ± 1.39	4.67 ± 1.49	0.458
Intact parathyroid hormone (pg/mL)	221.80 (98.83–474.63)	270.20 (122.00–433.55)	161.50 (69.20–548.73)	222.80 (92.65–714.20)	0.545
Urea reduction rate	0.72 ± 0.05	0.72 ± 0.05	0.73 ± 0.05	0.73 ± 0.05	0.625
Kt/V (Gotch)	1.30 ± 0.19	1.281 ± 0.18	1.32 ± 0.20	1.31 ± 0.19	0.531
ARB use, *n* (%)	59 (50.9)	29 (59.2)	22 (44.0)	8 (47.1)	0.301
β-blocker use, *n* (%)	30 (25.9)	12 (24.5)	13 (26.0)	5 (29.4)	0.923
CCB use, *n* (%)	45 (38.8)	20 (40.8)	17 (34.0)	8 (47.1)	0.589
α-adrenergic blockers, *n* (%)	17 (14.7)	6 (12.2)	10 (20.0)	61 (5.9)	0.299
Statin use, *n* (%)	33 (28.4)	14 (28.6)	15 (30.0)	4 (23.5)	0.877
Fibrate use, *n* (%)	9 (7.8)	6 (12.2)	2 (4.0)	1 (5.9)	0.294
Causes of hemodialysis					
Diabetes nephropathy, *n* (%)	54 (46.6)	23 (46.9)	26 (52.0)	5 (29.4)	0.272
Hypertensive nephrosclerosis, *n* (%)	31 (26.7)	13 (26.5)	13 (26.0)	5 (29.4)	0.962
Glomerulonephritis, *n* (%)	17 (14.7)	7 (14.3)	7 (14.0)	3 (17.6)	0.930
Others, *n* (%)	14 (12.1)	6 (12.2)	4 (8.0)	4 (23.5)	0.236
Vascular access					
Arteriovenous fistula, *n* (%)	91 (78.4)	39 (79.6)	40 (80.0)	12 (70.6)	0.696
Arteriovenous graft, *n* (%)	14 (12.1)	7 (14.3)	4 (8.0)	3 (17.6)	0.471
Hickman catheter, *n* (%)	11 (9.5)	3 (6.1)	6 (12.0)	2 (11.8)	0.572

Non-normally distributed variables are expressed as medians and interquartile range and tested using Kruskal–Wallis analysis; continuous variables are expressed as means ± standard deviation and tested using one-way analysis of variance. Categorical values are expressed as percentages and examined using the chi-square test. MHD, hemodialysis; ANGPTL3, angiopoietin-like protein 3; Kt/V, fractional clearance index for urea; ARB, angiotensin receptor blocker; CCB, calcium channel blocker. * Statistical significance was defined as *p* < 0.05.

**Table 2:** Correlation between clinical variables and the level of the vascular reactivity index.

Variables	Vascular Reactivity Index				
	Simple Regression		Multivariable Regression		
	*r*	*p* Value	Beta	Adjusted R^2^ Change	*p* Value
Age (years)	−0.132	0.157	—	—	—
Height (cm)	0.120	0.199	—	—	—
Pre-HD body weight (kg)	0.156	0.095	—	—	—
Post-HD body weight (kg)	0.156	0.095	—	—	—
Body mass index (kg/m^2^)	0.128	0.169	—	—	—
Log-HD duration (months)	−0.013	0.886	—	—	—
Systolic blood pressure (mmHg)	0.126	0.177	—	—	—
Diastolic blood pressure (mmHg)	0.133	0.155	—	—	—
Hemoglobin (g/dL)	0.024	0.796	—	—	—
Albumin (g/dL)	0.138	0.140	—	—	—
Total cholesterol (mg/dL)	−0.104	0.266	—	—	—
Log-Triglyceride (mg/dL)	0.065	0.490	—	—	—
Log-Glucose (mg/dL)	0.067	0.474	—	—	—
Log-ALP (U/L)	−0.187	0.045 *	—	—	—
Blood urea nitrogen (mg/dL)	0.134	0.151	—	—	—
Creatinine (mg/dL)	0.180	0.053	—	—	—
Total calcium (mg/dL)	−0.031	0.741	—	—	—
Phosphorus (mg/dL)	0.129	0.167	—	—	—
Log-iPTH (pg/mL)	−0.059	0.530	—	—	—
ANGPTL3 (ng/mL)	−0.319	<0.001 *	−0.319	0.094	<0.001 *
Urea reduction rate	−0.111	0.238	—	—	—
Kt/V (Gotch)	−0.119	0.204	—	—	—

Before being examined, the HD duration, lipid, glucose, ALP, and iPTH data were log-transformed due to their skewed distribution. Data were analyzed using both simple linear regression analysis and multivariable stepwise linear regression analysis (adapted factors were log-ALP and ANGPTL3). HD, hemodialysis; ALP, alkaline phosphatase; iPTH, intact parathyroid hormone; ANGPTL3, angiopoietin-like protein 3; Kt/V, fractional clearance index for urea. * *p* < 0.05 was considered statistically significant.

**Table 3:** The association between ANGPTL3 and vascular reactivity dysfunction and poor vascular reactivity.

Model	ANGPTL3 (per 1 ng/mL of Increase) for Vascular Reactivity Dysfunction		ANGPTL3 (per 1 ng/mL of Increase) for Poor Vascular Reactivity	
	OR (95% CI)	*p* Value	OR (95% CI)	*p* Value
Crude model	1.012 (1.003–1.021)	0.006 *	1.017 (1.007–1.027)	0.001 *
Adjusted model	1.015 (1.005–1.026)	0.005 *	1.024 (1.010–1.037)	0.001 *

Adjusted model: sex, age, body mass index, diabetes mellitus, hypertension, hemodialysis duration, systolic blood pressure, diastolic blood pressure, blood urea nitrogen, creatinine, total cholesterol, triglyceride, alkaline phosphatase, Kt/V, and ANGPTL3. ANGPTL3, angiopoietin-like protein 3; Kt/V, fractional clearance index for urea; OR, odds ratio; CI, confidence interval. * *p* < 0.05 was considered statistically significant.

**Table 4:** Diagnostic value of angiopoietin-like protein 3 on vascular reactivity dysfunction (intermediate vascular reactivity and poor vascular reactivity) or poor vascular reactivity.

**Vascular Reactivity Dysfunction**							
	**AUC (95% CI)**	***p* Value**	**Cut-Off**	**Sen (%)**	**Spe (%)**	**PPV (%)**	**NPV (%)**
ANGPTL3 (ng/mL)	0.635 (0.541–0.723)	0.0086	98.88	61.2	67.4	71.9	56.1
**Poor Vascular Reactivity**							
	**AUC (95% CI)**	***p* Value**	**Cut-off**	**Sen (%)**	**Spe (%)**	**PPV (%)**	**NPV (%)**
ANGPTL3 (ng/mL)	0.713 (0.622–0.793)	0.0019	158.18	41.2	91.9	24.4	91.0

ANGPTL3, angiopoietin-like protein 3; AUC, area under the curve; 95% CI, 95% confidence interval; Sen, sensitivity; Spe, specificity; PPV, positive predictive value; NPV, negative predictive value.

## Data Availability

The corresponding author can supply the study’s data upon request.
